# The impact of integrated genomic analysis on molecular classifications and prognostic risk stratification in endometrial cancer: a Chinese experience

**DOI:** 10.3389/fonc.2025.1541562

**Published:** 2025-02-06

**Authors:** Qian Zheng, Di Shao, Jin Shu, Qin Zhang, Min Huang, Dong Wang, Dongling Zou

**Affiliations:** ^1^ Department of Gynecologic Oncology, Chongqing University Cancer Hospital, Chongqing, China; ^2^ Chongqing Specialized Medical Research Center of Ovarian Cancer, Chongqing, China; ^3^ Organoid Transformational Research Center, Chongqing Key Laboratory of Translational Research for Cancer Metastasis and Individualized Treatment, Chongqing University Cancer Hospital, Chongqing, China; ^4^ BGI Genomics, Shenzhen, China

**Keywords:** endometrial cancer, molecular classification, next generation sequencing (NGS), adjuvant therapy, genetic tumor syndromes

## Abstract

**Background:**

The molecular classification of endometrial cancer (EC), as proposed by The Cancer Genome Atlas (TCGA), has transformed tumor classification, but there is a lack of extensive research on the molecular profiles and subtyping of endometrial cancer patients in China.

**Methods:**

200 EC patients were classified into the following four molecular types: (i) POLEmut; (ii) MSI-H; (iii) TP53mut; (iv) NSMP. This study aimed to investigate the molecular characteristics of EC patients at a single center by large-scale next generation sequencing(NGS), including clinicopathological features and gene mutations in patients with distinct molecular types, and to assess the relevance of molecular subtyping for postoperative adjuvant therapy.

**Results:**

NSMP group was the most prevalent, comprising 46.0% (92/200) of cases, followed by the TP53mut group at 17.5% (35/200), the MSI-H group at 23.5% (47/200), and the POLEmut group at 13.0% (26/200). CTNNB1 mutations were common in the POLEmut group but rare in the TP53mut group. With the application of the new European Society for Medical Oncology (ESMO) 2022 classification, 27 patients (14.1%) were reclassified. Concordance between the two classifications regarding postoperative risk was observed in 85.9% (165/192) of cases. Seven patients (3.6%) were downstaged, and twenty patients (10.4%) were upgraded. Additionally, the analysis revealed that eleven genes were significantly mutated in patients with lymphovascular space invasion (LVSI) compared to those without LVSI. Notably, NSD3 and POLD1 were highly mutated in patients with lymphatic metastasis compared to those without lymphatic metastasis. Conclusively, large-scale NGS has revolutionized EC management by facilitating rapid molecular subtype identification, guiding tailored adjuvant therapies, targeted treatments, and immunotherapies, and efficiently screening for Lynch syndrome, thereby significantly improving patient outcomes.

## Introduction

Endometrial cancer (EC) is a prevalent malignant tumor of the female reproductive tract, with rising incidence rates in China. In 2022, an estimated 84,520 new cases and 17,543 deaths occurred in China (1). Traditionally, the management of endometrial cancer has relied on conventional risk classification systems based solely on pathological factors such as tumor grade, stage, and depth of myometrial invasion (2). However, these traditional systems have been noted for their inter-observer variability and lack of reproducibility when establishing prognostic groups (2). The landscape of endometrial cancer management is evolving with the integration of molecular profiling, offering a more precise approach to risk assessment and treatment stratification (3, 4).

The Cancer Genome Atlas (TCGA) study has significantly reshaped our understanding of endometrial cancer by identifying distinct molecular subtypes with varying prognoses (5). This molecular classification has revolutionized risk assessment, providing a detailed framework for personalized treatment strategies that extend beyond conventional clinicopathologic factors (6). Furthermore, the development of improved molecular typing models, such as the Proactive Molecular Risk Classifier for Endometrial Cancer (ProMisE) and Translational PORTEC (TransPORTEC), has further refined risk classification by including DNA mismatch repair status, p53 mutations, and POLE mutations (7, 8). Molecular subtyping is crucial for guiding adjuvant therapy and prognosis prediction in high-risk and high-grade patients (9, 10).

With the maturation of molecular subtyping, major guidelines have incorporated it into prognosis risk assessment for patients with endometrial cancer. In 2020, the European Society of Gynaecological Oncology (ESGO), the European Society for Radiotherapy and Oncology (ESTRO), and the European Society of Pathology (ESP) (ESGO/ESTRO/ESP) first integrated molecular subtyping into their risk assessment for endometrial cancer. It provided stratification methods with or without molecular subtyping, allowing patients to choose postoperative risk levels based on whether molecular subtyping was performed (11). This guidance has been updated annually based on research progress. In 2022, the European Society of Medical Oncology (ESMO) published new clinical practice guidelines for endometrial cancer, recommending stratification methods that exclusively incorporate molecular subtyping (12). This update divides patients into low-risk, intermediate-risk, intermediate-high-risk, and high-risk groups and advises that clinical practice should follow these categories for treatment and follow-up. Consequently, in clinical practice, if a patient undergoes molecular subtyping, the ESMO 2022 guidelines will be used to assess prognosis risk levels and guide postoperative adjuvant therapy. While only the European guidelines recommend risk stratification based on molecular findings, National Comprehensive Cancer Network (NCCN) does not consider molecular biology to be a tool for defining prognostic groups and adjuvant therapy (2).

However, there are challenges associated with molecular typing. The NSMP (no specific molecular profile) group, characterized by a lack of distinctive gene alterations, exhibits substantial heterogeneity, necessitating further research to refine this phenotype (13, 14). Additionally, both the TCGA molecular typing and the subsequent ProMisE molecular typing programs are based on European and American data. Extensive studies are needed to determine their applicability to the Chinese population. Currently, both ProMisE and TransPORTEC classifications rely on two immunohistochemistry (IHC) tests and one gene sequencing test, which requires the use of multiple technologies in conjunction. This complexity poses a barrier to implementing ProMisE molecular classification in general pathology laboratories. Furthermore, IHC detection methods are susceptible to variability in human evaluation, which can lead to inconsistent results. For instance, approximately 15% of patients in the TP53mut group may be misclassified as belonging to the NSMP group when using the IHC method (15).

Advancements in Next Generation Sequencing (NGS) technology have significantly progressed the development of comprehensive molecular typing approaches. These advancements facilitate the analysis of multiple molecular alterations, including gene mutations, copy number variations, and gene expression profiles (10). This integrated molecular profiling enhances our understanding of tumor biology, establishes a foundation for identifying potential therapeutic targets, predicts responses to targeted therapies, and aids in diagnosing hereditary cancer syndromes such as Lynch syndrome (16).

Currently, there is a lack of extensive research on the molecular profiles and subtyping of endometrial cancer patients in China. This study represents the largest real-world analysis conducted in southwest China to date, aiming to comprehensively examine the molecular characteristics and related gene mutations of endometrial cancer patients in this region through NGS. The goal is to provide precise guidance for postoperative adjuvant therapy, predict potential treatment targets, and offer genetic counseling.

## Materials and methods

### Study design and participants

This retrospective study analyzed 200 newly diagnosed endometrial cancer patients admitted to the Gynecological Cancer Center of Chongqing University Cancer Hospital from August 2020 to January 2023. We collected and organized patients’ basic information, pathological reports, treatment processes, and outcomes. Based on the choices of physicians and patients, tumor molecular analyses were performed using two approaches: 1) 124 cases underwent comprehensive germline and somatic genetic testing, with their tumor tissues (formalin-fixed paraffin-embedded [FFPE]) and peripheral blood samples subjected to tumor-normal matching NGS of 1021 tumor-related genes; 2) POLE, MSI, and TP53 genes were detected in FFPE samples from 76 cases using NGS.

### NGS comprehensive genomic profiling and molecular classification

Genomic DNA (200 ng) was fragmented, and library construction was performed using CoBox adaptors, a proprietary design by BGI Genomics Co., Ltd., incorporating Unique Molecular Identifiers (UMI) and dual indexing. This design effectively reduces background noise and ensures accurate detection of genetic variations. Libraries, with a total DNA of 1 μg, were hybridized to custom-designed biotinylated oligonucleotide probes (Integrated DNA Technology) covering 688 genes, including those linked to hereditary cancer risk, DNA repair pathways, driver genes, and tumor suppressor genes associated with gynecological tumors. Enriched DNA samples were sequenced using 100-bp paired-end reads on the MGISEQ-2000 platform (MGI Tech).

The sequencing data underwent preprocessing, including the removal of terminal adaptor sequences and low-quality reads. Clean reads were aligned to the reference human genome (hg19) using BWA (version 0.7.12). PCR duplicates were marked using Picard (version 1.98), and realignment and recalibration were performed with GATK (version 4.0). Germline and somatic single nucleotide variants (SNVs) and small insertions and deletions (Indels) were called using GATK HaplotypeCaller and MuTect2 (version 4.0). Somatic copy-number alterations were identified using CONTRA (v2.0.8), and structural variations were detected using in-house software. Single nucleotide/indel variants were annotated and filtered with Ensembl Variant Effect Predictor (ensembl-vep 90.6). Microsatellite instability (MSI) status was inferred using MSIsensor software, and distribution differences among microsatellite fragments were analyzed using MANTIS software. Tumor mutation burden (TMB) was calculated as the number of non-synonymous mutations in non-driver genes per sample divided by the genomic coverage for that sample.

Tumors were assigned hierarchically to four molecular subtypes, paralleling those described by TCGA (5). NGS was used to detect the molecular characteristics of the tumors and classify patients into one of the following four molecular subtypes: (i) polymerase epsilon exonuclease domain ultramutated (POLEmut), characterized by 11 well-characterized disease-causing mutations (17); (ii) microsatellite instability hypermutated (MSI-H), with a high mutational frequency (>10 mutations/Mb); (iii) TP53mut group, marked by high somatic copy number alterations, low mutation rate, and TP53 mutations; (iv) NSMP (no specific molecular profile), encompassing tumors without the aforementioned genetic alterations.

### Prognostic risk grouping and treatment

All patients undergoing surgery were classified into different risk levels based on the ESGO/ESTRO/ESP 2020 guidelines (11) without considering molecular results, as well as the ESMO 2022 guidelines (12), which incorporated molecular subtyping (see **Supplementary Table 1**). Notably, treatment decisions were based on the risk group classification, including molecular classification, recommended by the most recent guidelines at the time.

### Statistical analysis

Data were collected on a secure Excel sheet. Summary statistics were reported as numbers or as mean ± standard deviation. Group comparisons were performed using the χ² test, t-test, Fisher’s exact test, Kruskal–Wallis test, or Mann–Whitney test, as appropriate. All reported p-values were based on two-sided tests, with a significance level of p < 0.05. Statistical analyses were conducted using R software (version 4.2.1).

## Results

### Patient characteristics

The study included 200 patients with endometrial cancer, all of whom provided qualified tumor samples for molecular analysis. The main patient characteristics are presented in **Table 1**. The median age at diagnosis was 53.4 years (range: 28.0–80.0), and the median Body Mass Index (BMI) was 25.5 kg/m². The majority of cases (190, 95.5%) were classified as endometrioid histotype, 4 cases (2.0%) were clear cell, and the remaining cases included serous, sarcoma, and mixed histologies. Regarding tumor grade, 48 cases (25.9%) were grade 1, 90 cases (48.6%) were grade 2, and 47 cases (25.4%) were grade 3. According to the International Federation of Gynecology and Obstetrics (FIGO) stage, 108 patients (54.3%) were diagnosed with stage IA disease, while the rest were distributed as 25 (12.6%) stage IB, 29 (14.6%) stage II, 28 (14.1%) stage III, and 9 (4.5%) stage IV. Lymphovascular space invasion (LVSI) assessment in 189 cases revealed 41 cases (21.7%) positive for LVSI according to the ESMO 2022 criteria. Postoperative pathology indicated pelvic lymph node involvement in 5.2% of patients (10 out of 191) and para-aortic lymph node positivity in 3.2% (6 out of 191).

**Table 1:** Clinicopathologic and molecular characteristics and prognostic risk stratification of the study population by molecular subtypes.

	*POLE*mut (N=26)	MSI-H (N=47)	*TP53*mut (N=35)	NSMP (N=92)	Total (N=200)	p value
Age						0.251
Mean (SD)	54.6 (11.8)	54.8 (7.0)	54.1 (11.4)	52.0 (8.0)	53.4 (9.1)	
Range	28.0 - 80.0	41.0 - 78.0	36.0 - 73.0	28.0 - 80.0	28.0 - 80.0	
BMI						0.469
Mean (SD)	24.3 (4.3)	24.3 (3.5)	28.2 (27.6)	25.3 (3.7)	25.5 (11.9)	
Range	16.4 - 40.0	17.0 - 32.0	9.6 - 34.1	15.5 - 36.0	9.6 - 40.0	
Histological Type						0.187
Endometroid	26 (100.0%)	47 (100.0%)	29 (85.3%)	88 (95.7%)	190 (95.5%)	
Clear Cell	0 (0.0%)	0 (0.0%)	2 (5.9%)	2 (2.2%)	4 (2.0%)	
Sarcoma	0 (0.0%)	0 (0.0%)	3(8.8%)	1 (1.1%)	4 (2.0%)	
Serous	0 (0.0%)	0 (0.0%)	0 (0.0%)	1 (1.1%)	1 (0.5%)	
NA	0	0	1	0	1	
FIGO Stage						0.002
Ia	17 (65.4%)	20 (42.6%)	12 (35.3%)	59 (64.1%)	108 (54.3%)	
Ib	3 (11.5%)	9 (19.1%)	4 (11.8%)	9 (9.8%)	25 (12.6%)	
II	1 (3.8%)	11 (23.4%)	4 (11.8%)	13 (14.1%)	29 (14.6%)	
III	5 (19.2%)	4 (8.5%)	9 (26.5%)	10 (10.9%)	28 (14.1%)	
IV	0 (0.0%)	3 (6.4%)	5 (14.7%)	1 (1.1%)	9 (4.5%)	
NA	0	0	1	0	1	
Grade						0.003
G1	4 (15.4%)	10 (21.7%)	6 (22.2%)	28 (32.6%)	48 (25.9%)	
G2	13 (50.0%)	21 (45.7%)	8 (29.6%)	48 (55.8%)	90 (48.6%)	
G3	9 (34.6%)	15 (32.6%)	13 (48.1%)	10 (11.6%)	47 (25.4%)	
NA	0	1	8	6	15	
LVSI						0.030
Yes	5 (20.8%)	16 (35.6%)	8 (25.8%)	12 (13.5%)	41 (21.7%)	
No	19 (79.2%)	29 (64.4%)	23 (74.2%)	77 (86.5%)	148 (78.3%)	
NA	2	2	4	3	11	
Myometrial Invasion						0.005
<50%	14 (58.3%)	23 (50.0%)	22 (73.3%)	66 (73.3%)	125 (65.8%)	
>=50%	6 (25.0%)	21 (45.7%)	8 (26.7%)	15 (16.7%)	50 (26.3%)	
None	4 (16.7%)	2 (4.3%)	0 (0.0%)	9 (10.0%)	15 (7.9%)	
NA	2	1	5	2	10	
Residual Tumor						0.095
Yes	0 (0.0%)	2 (4.3%)	0 (0.0%)	0 (0.0%)	2 (1.0%)	
No	26 (100.0%)	45 (95.7%)	31 (100.0%)	91 (100.0%)	193 (99.0%)	
NA	0	0	4	1	5	
Nerve Invasion						0.370
Yes	0 (0.0%)	1 (2.2%)	1 (3.3%)	0 (0.0%)	2 (1.1%)	
No	23 (100.0%)	44 (97.8%)	29 (96.7%)	86 (100.0%)	182 (98.9%)	
NA	3	2	5	6	16	
Lymphatic Metastasis						0.012
Negative	23 (92.0%)	41 (91.1%)	24 (77.4%)	87 (96.7%)	175 (91.6%)	
Paraaortic	1 (4.0%)	2 (4.4%)	2 (6.5%)	1 (1.1%)	6 (3.2%)	
Pelvic Cavity	1 (4.0%)	2 (4.4%)	5 (16.1%)	2 (2.2%)	10 (5.2%)	
NA	1	2	4	2	9	
Diagnostic peritoneal lavage						0.094
Negative	23 (100.0%)	36 (85.7%)	18 (75.0%)	72 (86.7%)	149 (86.6%)	
Positive	0 (0.0%)	6 (14.3%)	6 (25.0%)	11 (13.3%)	23 (13.4%)	
NA	3	5	11	9	28	
TMB						< 0.001
Mean (SD)	147.7 (151.1)	31.6 (19.6)	4.7 (7.1)	4.6 (6.3)	31.5 (75.5)	
Range	43.3 - 643.3	3.5 - 77.4	0.1 - 30.4	0.1 - 34.0	0.1 - 643.3	
ESMO 2022 prognostic risk group						< 0.001
NA	2	0	4	2	8	
High	5 (20.8%)	8 (17.0%)	31 (100.0%)	13 (14.4%)	57 (29.7%)	
Intermediate	0 (0.0%)	8 (17.0%)	0 (0.0%)	16 (17.8%)	24 (12.5%)	
Intermediate High	0 (0.0%)	15 (31.9%)	0 (0.0%)	11 (12.2%)	26 (13.5%)	
low	19 (79.2%)	16 (34.1%)	0 (0.0%)	50 (55.6%)	85 (44.3%)	

BMI, median Body Mass Index; NA, not available; FIGO, International Federation of Gynecology and Obstetrics; LVSI, lymphovascular space invasion; TMB, tumor mutation burden; ESMO, European Society for Medical Oncology.

### Molecular classification and genomic profile

**Figure 1:**
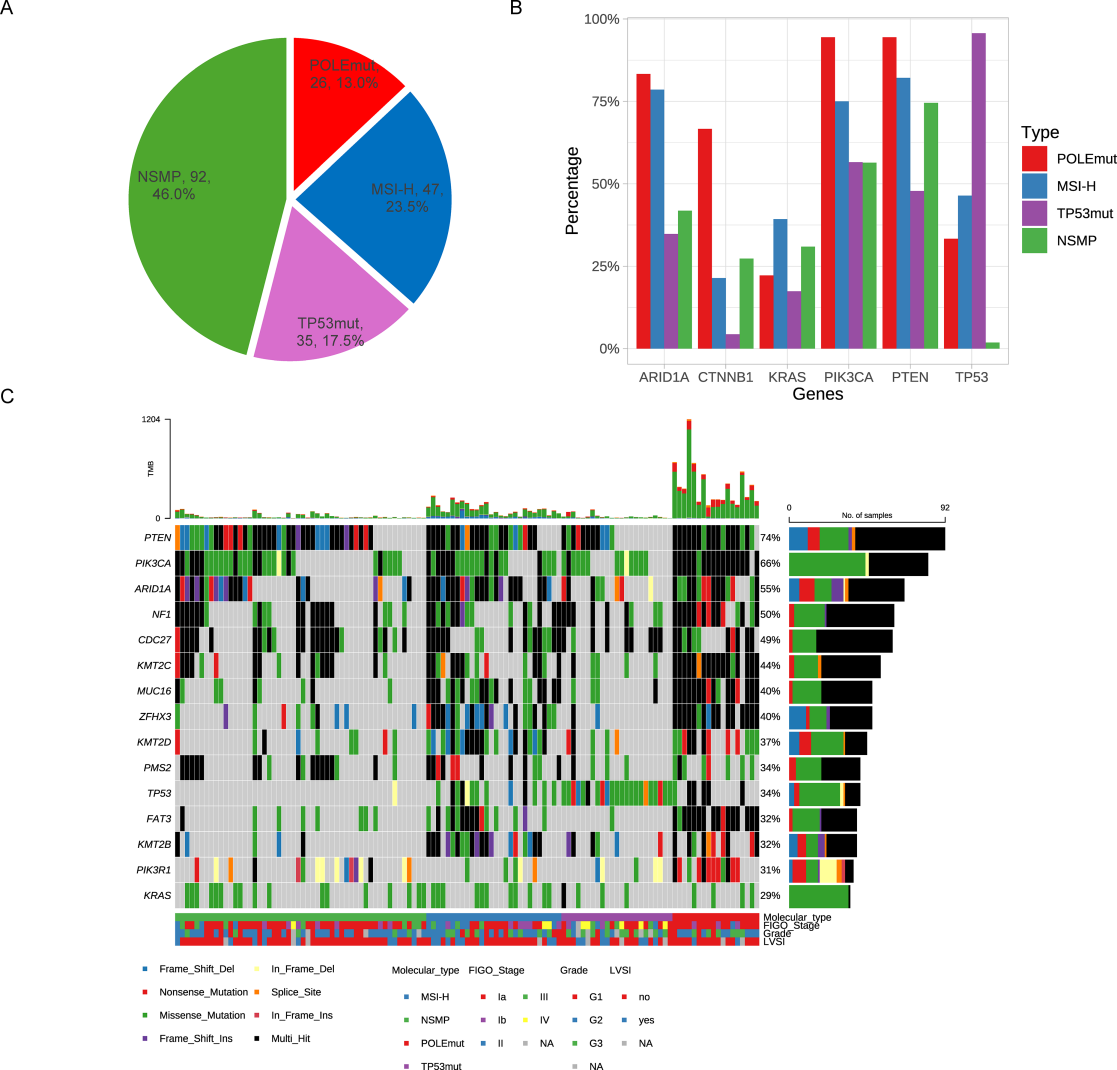
Molecular classification and genomic profiles of patients with endometrioid carcinoma. **(A)** Distribution of molecular subtypes **(B)** Mutation rate of high-frequency mutated genes in each molecular typing **(C)** Genomic and histopathologic characterization of each molecular type.

**Figure 2:**
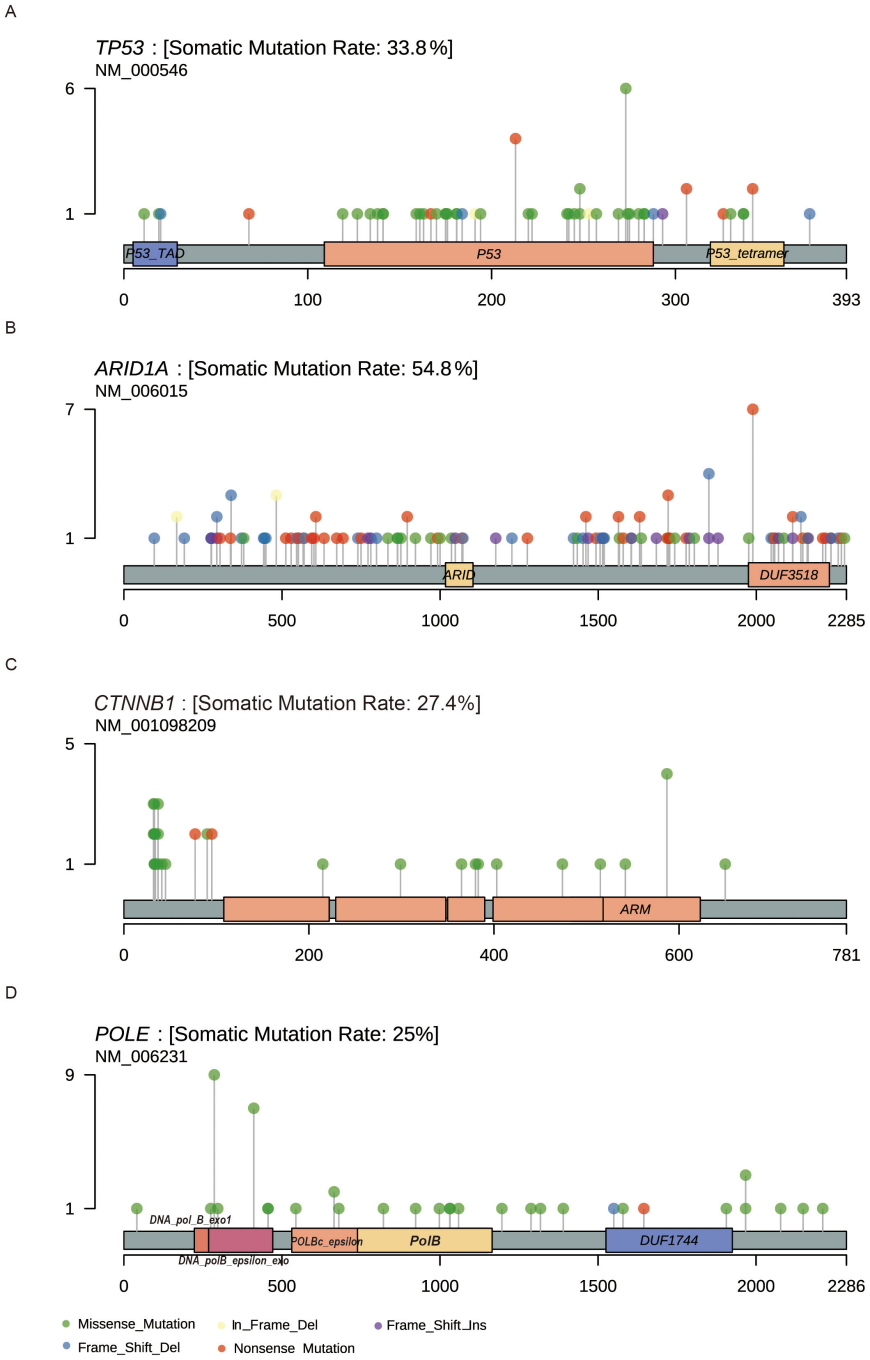
Lollipop plots showing the location of the amino acid change in driver genes. **(A)** TP53 **(B)** ARID1A **(C)** CTNNB1 **(D)** POLE.

Of the 200 eligible patients, 26 (13.0%) were classified as POLEmut, 47 (23.5%) as MSI-H, 35 (17.5%) as TP53mut, and 92 (46.0%) as NSMP (**Figure 1A**). Additional details on the spectrum of POLE exonuclease domain mutations (EDMs) and TP53 mutations identified are provided in **Figures 2A, D**, respectively. A small proportion of patients (1.5%) exhibited multiple molecular features: two POLEmut tumors had either a TP53 mutation or were MSI-H, and one MSI-H tumor had a TP53 mutation. According to the ESMO 2022 guidelines, which classify patients based on POLE mutation status, MSI status, and TP53 mutation, these cases were categorized as POLEmut or MSI-H accordingly.

We analyzed the associations between molecular subtypes and clinicopathological parameters (**Table 1**). All tumors classified as POLEmut were endometrioid carcinoma, primarily stage I, with an ultra-high TMB and a low rate of lymph node metastasis. MSI-H subtype tumors exhibited aggressive traits, including lymphovascular space invasion (LVSI) and myometrial invasion, and had the second-highest TMB. The TP53mut group was characterized by high-grade tumors at advanced stages, with a higher prevalence of myometrial invasion and lymphatic metastasis. The NSMP group comprised the largest proportion of endometrial cancers, predominantly microsatellite-stabilized (MSS) tumors lacking distinct clinicopathological features.

We examined the mutation profiles across the four subtypes (**Figure 1**). The most frequently mutated genes in the overall population were PTEN, PIK3CA, ARID1A, NF1, and CDC27 (**Figure 1C**). Notably, PTEN mutations often co-occurred with PIK3CA mutations, while ARID1A mutations were rarely observed in the TP53mut subgroup **(Figure 1B**). In the NSMP subgroup, the mutation rates were as follows: PTEN (75%), PIK3CA (56%), CDC27 (45%), ARID1A (42%), and NF1 (40%) (**Supplementary Figure 1**).

**Figure 3:**
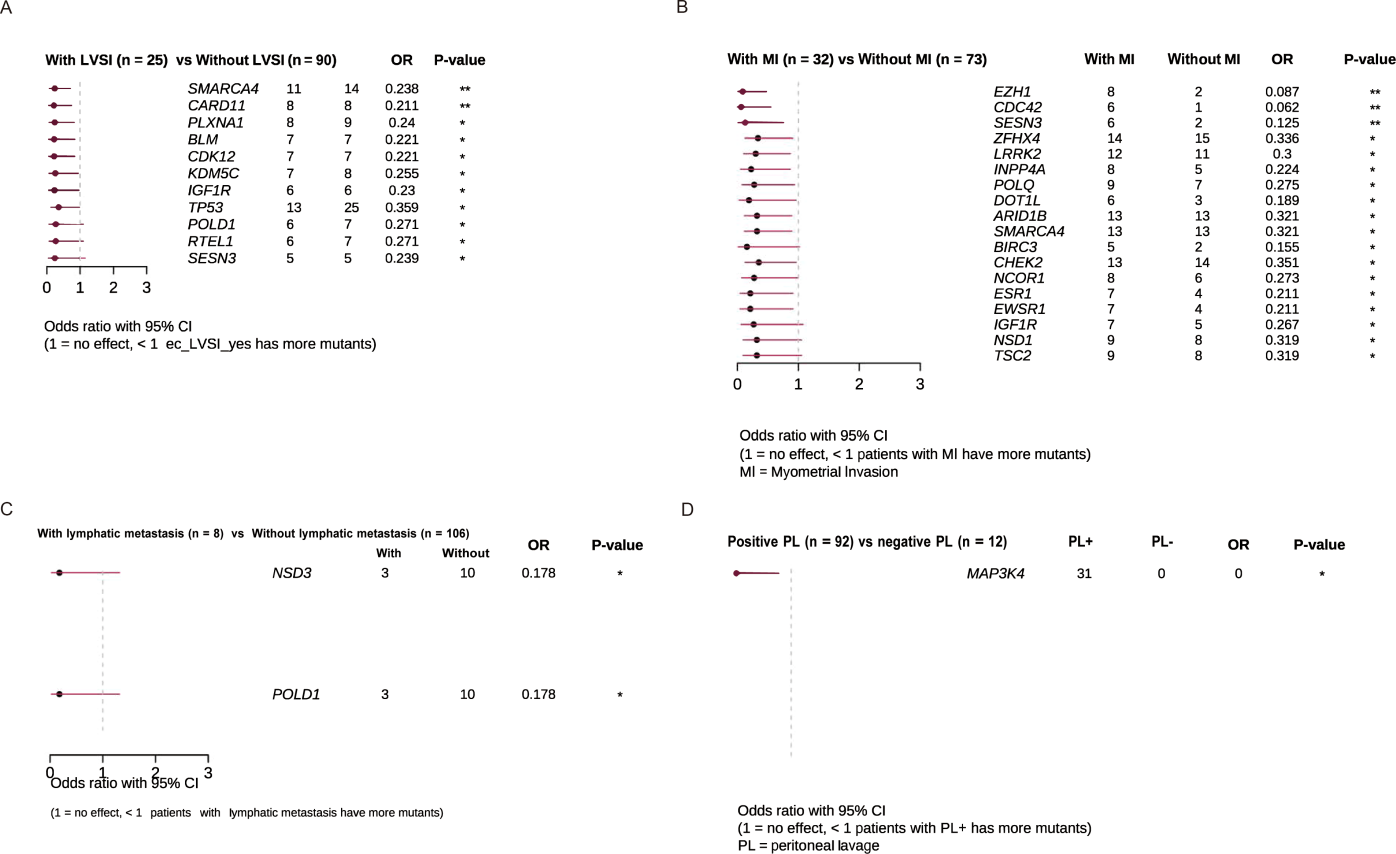
Clinical enrichment analysis identified enriched mutations associated with. **(A)** LVSI **(B)** Myometrial invasion **(C)** Lymphatic metastasis **(D)** Peritoneal lavage.

We analyzed high-frequency mutated genes associated with key risk factors in the stratification of endometrial cancer, including LVSI, myometrial invasion, positive peritoneal cytology, and lymph node metastasis. LVSI-positive patients exhibited a higher mutation burden in genes such as SMARCA4, CARD11, PLXNA1, BLM, and CDK12 (**Figure 3A**). These genes are involved in critical signaling pathways, including the Wnt/β-catenin pathway, NF-κB signaling pathway, and semaphorin signaling pathway. Patients with myometrial invasion displayed a notable increase in mutations in genes such as EZH1, CDC42, SESN3, ZFHX4, LRRK2, INPP4A, and POLQ compared to those without myometrial invasion (**Figure 3B**). However, no significant enrichment of gene mutations was observed in association with lymph node metastasis or positive peritoneal cytology (**Figures 3C, D**).

Additionally, we examined the distribution of mutations in key cancer genes. TP53 mutations were distributed throughout the gene without distinct mutation hotspots, with missense and nonsense mutations being the primary types (**Figure 2A**). Although ARID1A mutations appeared relatively random, a hotspot mutation (Arg1989Ter) was identified in 7 patients (**Figure 2B**). CTNNB1 mutations were common in the POLEmut group but rare in the TP53mut group (**Figure 2C**). Consistent with prior research, we identified hotspot mutations in the POLE gene at Pro286Arg and Val411Leu, found in 9 (34.6%) and 7 (26.9%) of the 26 POLEmut samples, respectively (**Figure 2D**).

### Risk reclassification after incorporating molecular typing

We compared the ESMO 2022 classification, which incorporates molecular typing, with the ESGO 2020 classification, which does not include molecular typing. As a result, 27 patients (14.1%) were reclassified after applying the new ESMO 2022 guidelines (**Table 2**). Concordance between the two classifications in terms of postoperative risk was 85.9% (165/192). In this study, seven patients (3.6%) were downstaged, and twenty patients(10.4%) were upgraded.

**Table 2:** Number of patients classified into risk groups according to ESGO/ESTRO/ESP 2020 guidelines and ESMO 2022 guidelines recommendations.

ESGO 2020	Low (N=88)	Intermediate (N=30)	Intermediate High (N=37)	High and metastatic (N=37)	Total (N=192)
ESMO 2022					
Low	80 (90.9%)	1 (3.3%)	4 (10.8%)	0 (0.0%)	85 (45.2%)
Intermediate	0 (0.0%)	22 (73.3%)	2 (5.4%)	0 (0.0%)	24 (12.8%)
Intermediate High	0 (0.0%)	0 (0.0%)	26 (70.3%)	0 (0.0%)	26 (13.8%)
High	8 (9.1%)	7 (23.3%)	5 (13.5%)	37 (100.0%)	57 (28.2%)

ESGO/ESTRO/ESP, The European Society of Gynaecological Oncology (ESGO), the European Society for Radiotherapy and Oncology (ESTRO) and the European Society of Pathology (ESP); ESMO, European Society for Medical Oncology.

**Figure 4:**
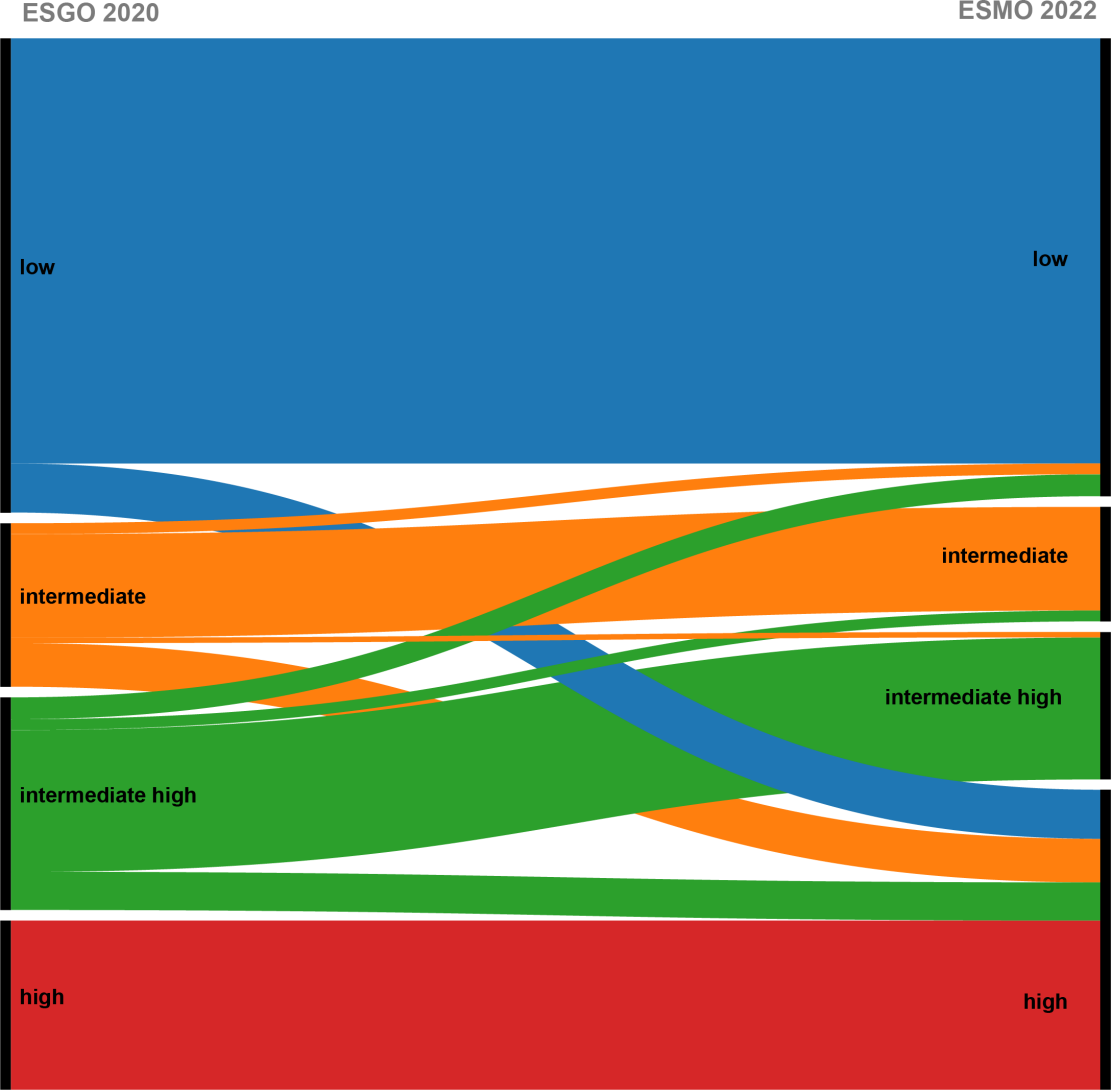
Shift of patients after updating the risk model following the addition of molecular typing.

In the study, 20 (10.4%) stage I-II patients were reclassified into the high-risk group in accordance with the ESMO 2022 guidelines due to TP53 mutations, including 8 patients in the ESGO 2020 low-risk group, 7 patients in the ESGO 2020 intermediate-risk group, and 5 patients in the ESGO 2020 intermediate-high-risk group. Conversely, a total of 7 (3.6%) early-stage patients were downshifted to lower risk categories: 1 patient was downgraded from the intermediate-risk group as defined by ESGO 2020 criteria to the low-risk group as per ESMO 2022 guidelines due to the presence of POLEmut, 4 patients were reassigned from the ESGO 2020 intermediate-high-risk group to the ESMO 2022 low-risk group, also attributed to POLEmut, and 2 patients were recategorized from the ESGO 2020 intermediate-high-risk group to the ESMO 2022 intermediate-risk group, owing to a refined assessment of tumor differentiation. The class alterations due to molecular typing are depicted in **Figure 4**.

From the above results, the changes in risk stratification included in molecular typing are mainly reflected in POLEmut type and TP53mut type. Patients with TP53mut and muscle-layer invasion at all stages and grades were upgraded to the high-risk group, with a total of 20 patients upgraded from various groups to high risk, thereby preventing undertreatment. Conversely, FIGO stage I–II POLEmut patients were downgraded to the low-risk group, with 7 patients being downgraded to avoid overtreatment. Notably, risk stratification changes were observed only in early-stage EC patients (stage I–II), while the stratification for patients with stage III–IV remained unchanged.

### Treatment

In our study, 4 of the 200 patients did not undergo surgical treatment, while 196 did. of these 196 patients, most underwent total hysterectomy and bilateral salpingo-oophorectomy (TH/BSO) along with pelvic lymph node dissection (180 patients, 91.8%). Eleven patients (5.6%) had TH/BSO and sentinel lymph node dissection, and 2 patients (1.0%) underwent hysteroscopic surgery to preserve fertility. Among the 193 patients who underwent radical surgery, 119 (61.6%) received postoperative adjuvant therapy: 63 (32.6%) received chemoradiotherapy, 48 (24.9%) received radiotherapy, and 8 (4.1%) received chemotherapy (**Table 3**).

**Table 3:** Surgical and adjuvant treatment of patients with endometrial cancer.

Surgery	N=200
TH/BSO	3 (1.6%)
TH/BSO+LND	180 (91.8%)
TH/BSO+SLN	11 (5.6%)
Hysteroscopy	2 (1.0%)
None	4

Adjuvant therapy	No. 200
Chemoradiotherapy	63 (32.6%)
Chemotherapy	8 (4.1%)
None	74 (38.4%)
Radiotherapy	48 (24.9%)
NA	7

TH/BSO, total hysterectomy and Bilateral salpingo-oophorectomy; LND, Lymph node dissection; SLN, Sentinel lymph node dissection; NA, not available.

Twenty patients upgraded from different ESGO 2020 risk groups to the high-risk group according to ESMO 2022 received standard surgical treatment and adjuvant chemoradiotherapy. None of the patients in our cohort were undertreated. Conversely, five patients downgraded from various ESGO 2020 risk groups to the ESMO 2022 low-risk group proceeded directly to follow-up after receiving standard surgical treatment.

## Discussion

Since the introduction of molecular classification for endometrial cancer in 2013, it has been incorporated into the WHO tumor classification and the guidelines of ESMO (12), ESGO/ESTRO/ESP (11), and NCCN (18). In 2021, China published an expert consensus on molecular typing for endometrial cancer. This study aimed to analyze gene mutations and molecular subtypes in endometrial cancer patients from a single center in Southwest China, focusing on treatment guidance and genetic counseling. A total of 200 patients were enrolled, with 124 undergoing comprehensive germline and somatic genetic testing. Most patients were diagnosed with endometrioid histotypes, showing varied disease grading and staging. Molecular classification identified four primary subtypes: POLEmut (13.0%), MSI-H (23.5%), TP53mut (17.5%), and NSMP (46.0%). The POLEmut subtype was associated with favorable clinicopathological features, while the TP53mut subtype was linked to more aggressive disease characteristics and advanced high-grade tumors. The most frequently mutated genes were PTEN, PIK3CA, ARID1A, NF1, and CDC27. Specific gene mutations were correlated with risk factors such as LVSI, peritoneal metastasis, and lymph node metastasis.

The distribution of molecular subtypes in this study shows some variation from the TCGA molecular classification scheme, which reports the proportions of POLE-mutant, MSI, copy number low (CNL), and TP53mut subtypes as 7%, 28%, 39%, and 26%, respectively (5). In this study, the proportion of POLEmut patients (13.0%) is comparable to that reported in India (10.4%) (19). The proportion of NSMP patients (46.0%) in this study is higher compared to TCGA (39%) and is similar to findings in Korea (58.8%) (20) and Thailand (52.9%) (21). However, Japan (38.6%) (22) and India (41.7%) (19) report NSMP proportions comparable to TCGA. The proportion of TP53mut patients in this study (17.0%) is lower compared to TCGA (26%) and also lower than that reported in Korea (16.2%) (20), Thailand (13.8%) (21) and Japan (8.7%) (22). In contrast, India (25%) (19) shows a proportion similar to TCGA. These findings highlight differences in molecular subtypes between Asian and Western populations, as well as among different Asian countries. Further research is needed to explore these differences in molecular subtypes and prognoses across Asian populations and countries. The PROBEAT study (NCT05179447), a randomized Phase III trial initiated on January 24, 2022, aims to evaluate adjuvant therapy tailored for Chinese endometrial cancer patients based on WHO-recognized molecular classification. The study plans to recruit 590 endometrioid endometrial cancer patients from 13 clinical centers in China, which will provide valuable data on the Chinese population (23).

In this study, seven patients were identified with germline pathogenic mutations in MLH1, MSH2, MSH6, PMS2, and EPCAM, confirming the diagnosis of Lynch syndrome (24). Of these, three had a family history of tumors. Notably, not all seven patients were classified as MSI-H; one patient was classified into the POLEmut group due to concurrent mutations in POLE, MSH6, and TP53 genes. This case represents a “triple classifier” endometrial cancer with MMRd, POLEmut, and TP53mut characteristics. In such instances, TP53 mutations are secondary events, and p53 immunohistochemistry alone has limited utility in distinguishing among these classifications. Determining whether POLE or MMRd are the primary drivers remains challenging with the current data. These triple-classifiers might be classified as POLEmut endometrial cancers if they carry a pathogenic POLE exonuclease domain mutation (EDM) as identified through whole-exome sequencing (WES) data (25). In patients with multiple classifier endometrial cancer, the presence of POLE mutations is associated with a favorable prognosis, and no recurrences were observed even when MMRd/MSI-H and/or p53 abnormalities coexist (26).

In addition to molecular typing, high-frequency mutant genes such as PTEN, KRAS, PIK3CA, and CTNNB1 have been identified. The significance of these auxiliary typing markers is becoming increasingly apparent with advancing research. In our study, the most frequently mutated genes were PTEN (74%), PIK3CA (66%), and ARID1A (55%). Specific gene mutations associated with high-risk factors, including LVSI and peritoneal metastasis, were enriched in genes involved in signaling pathways like SMARCA4, CARD11, PLXNA1, BLM, and CDK12. An increased frequency of mutations in genes such as EZH1, CDC42, SESN3, ZFHX4, LRRK2, INPP4A, and POLQ was observed in patients with peritoneal metastasis. These findings, not previously reported in other studies, underscore the potential of specific gene mutations to enhance risk stratification and guide treatment planning. For instance, patients with LVSI positive tumors might benefit from more intensive adjuvant therapies to mitigate the risk of recurrence.

While our study didn’t uncover a significant link between specific gene mutations and lymph node metastasis or positive peritoneal cytology, promising findings have been reported in several research. Mairé M et al. have reported that MSX1 gene expression is downregulated in patients with lymph node positivity, while FAP and ACTGA are upregulated (27). Additionally, another study identified a a panel of fivegenes (ASRGL1, RHEX, SCGB2A1, SOX17, and STX18) as biomarkers for predicting lymph node metastasis in early-stage endometrial cancer patients (28). Of course, other clinical factors, such as Ca-125 levels, thrombocytosis, and imaging results, also have some impact on lymph node metastasis risk and can improve preoperative risk stratification (29). On the other hand, molecular classification offers a more comprehensive assessment for endometrial cancer patients with lymph node metastasis. Schivardi G, et al. found that POLE mutated tumors are extremely rare in these patients, with NSMP being the largest subgroup (30). Chacon E, et al. showed significant differences in sentinel lymph node (SLN) involvement among different molecular subtypes in early-stage endometrial cancer patients, with the highest involvement rates in p53abn and MMRd groups at 12.50% and 12.40%, respectively (31). This underscores the importance of considering molecular features for accurate staging and optimizing patient management decisions.

There is notable variability in the management of EC, especially regarding the use of adjuvant therapy following hysterectomy (32). The ESGO/ESTRO/ESP 2020 guidelines recommend incorporating molecular subtypes, when available, into risk group assignments and adjuvant treatment recommendations. As the inclusion population is up to January 2023, the ESMO 2022 guidelines are applied. In clinical practice, molecular subtyping results are used according to the ESMO 2022 guidelines to assess prognosis and guide postoperative adjuvant therapy. Consistent with our findings, research shows that approximately 6-32.7% of patients experience risk group migration when using a classification system that incorporates molecular features compared to one based solely on clinicopathologic characteristics (33–36). Specifically, pathogenic POLE mutations often result in downgrading of the risk category, while TP53 mutations lead to an upgrade. This underscores the prognostic significance of molecular classification for accurate risk categorization and treatment planning (37). In our study, a higher rate of risk category upgrading was observed compared to other studies, which generally report a higher rate of downgrading (33, 38). This difference may be attributed to the frequent upgrading of patients with TP53 mutations detected in early-stage disease, which was relatively more common in our cohort. Although our study reports a lower proportion of TP53mut patients compared to the TCGA database (17.5% vs. 26%), the ESMO guidelines for postoperative risk stratification and adjuvant therapy align more closely with the ProMisE and Trans-PORTEC classifications. These classifications differ from TCGA’s molecular subtypes. Given that most of our patients were in the early stages, our study shows a higher proportion of TP53mut patients compared to the early-stage cohort in the Trans-PORTEC classification (15.5% vs. 9%) (8). This discrepancy contributes to the higher rate of risk category upgrading observed in our study. Preliminary results from the prospective PORTEC-3 trial suggest that molecular classification has strong prognostic value for high-risk EC, irrespective of tissue type, and advocate for reducing adjuvant therapy for POLEmut tumors (9).

Although NGS testing for large panels can lead to increased costs, multi-targeting can lead to more matched treatment regimens, and matched therapy can improve the prognosis of patients with malignancies (39, 40). In a prospective analysis, 47% (16/34) of EC patients matched for treatment after NGS combinatorial tumour analysis achieved clinical benefit, including 40% (2/5) of MSI-H patients treated with an immune checkpoint inhibitor and 42% (8/19) of patients matched on the basis of PIK3CA and PTEN mutations (41). Of the 1,281 U.S. physicians surveyed in 2017, three-quarters reported using NGS tests to guide treatment decisions (42). As China’s economy continues to grow, healthcare expenses for individuals are rising. The “Statistical Bulletin on the Development of China’s Health Care Industry in 2022” reported that the total national health expenditure reached 675.19 billion yuan in 2022, with per capita health expenditure at 6010 yuan. Large-panel genetic testing, now covered by medical insurance in the southwestern region of China, offers a cost-effective option for patients. This testing reduces financial burdens and provides a comprehensive assessment of genetic information, which is crucial for accurate molecular subtyping and personalized treatment plans. It helps avoid errors in immunohistochemistry, guides adjuvant therapy decisions, and supports the development of tailored treatment and genetic counseling strategies based on individual gene mutations.

This study is subject to several important limitations that should be considered when interpreting the findings. Firstly, the retrospective and single-institution design of this analysis may limit the generalizability of the results. As a single-center study, the patient population may not be fully representative of the broader endometrial cancer demographics in China, and differences in referral patterns, treatment approaches, and access to molecular testing at other institutions could lead to variations in the distribution of molecular subtypes. Additionally, potential source of bias was the heterogeneity in the depth of molecular characterization, with only 124 of the 200 patients undergoing the full NGS panel analysis. Meanwhile, the relatively recent introduction of comprehensive molecular profiling precluded the evaluation of long-term survival outcomes. Future research should aim to address these limitations through larger, multi-center prospective studies with long-term follow-up. This would enable a more comprehensive evaluation of the clinical implications of molecular subtyping in the Chinese endometrial cancer population.

## Conclusions

This study enhances the understanding of the integration of molecular profiling into the clinical management of endometrial carcinoma. By identifying molecular subtypes, gene mutations, and their associations with clinicopathological features, it provides a foundation for personalized treatment strategies, risk stratification, and prognostic assessment. Compared to previous classification methods, the ESMO 2022 classification combines molecular characteristics with clinical features, offering patients a more accurate risk assessment system. This approach is helpful to prevent overtreatment, reduce unnecessary treatment and alleviate financial burdens. Further research and validation studies are needed to translate these findings into clinical practice and improve patient outcomes in endometrial carcinoma.

## Data Availability

The original contributions presented in the study are included in the **Supplementary Material**, further inquiries can be directed to the corresponding authors.
