# Supplementary material for: The impact of integrated genomic analysis on molecular classifications and prognostic risk stratification in endometrial cancer: a Chinese experience
Source: Front Oncol. 2025 Feb 6;15:1541562. doi: 10.3389/fonc.2025.1541562 (PMC11839450; doi:10.3389/fonc.2025.1541562)
Supplement: Supplementary file 1 [file Table1.docx]

Supplementary Material

**
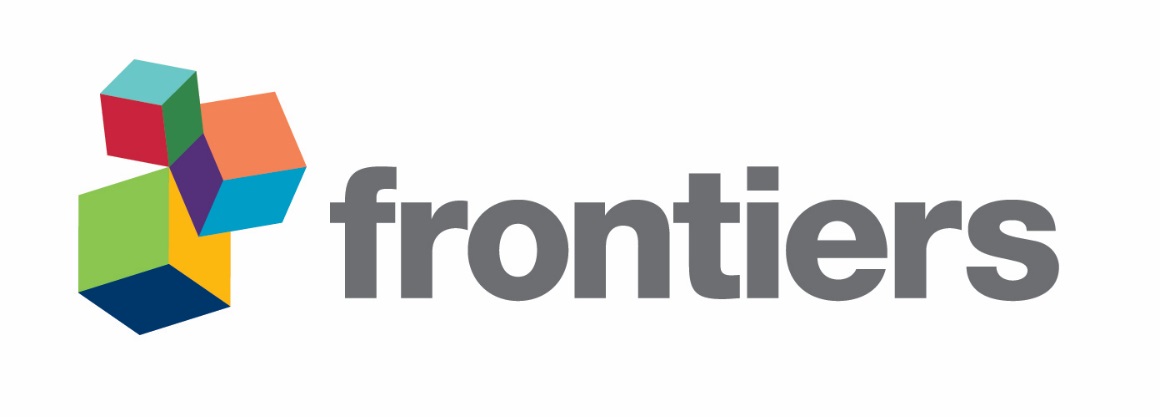
**

**Supplementary Table 1.** Risk Class Systems According to ESGO 2020 and ESMO 2022

| **Risk group** | **2020ESGO/ESTRO/ESP**  **Molecular classification unknown** | **2020ESGO/ESTRO/ESP**  **Molecular classification known** | **2022ESMO** |
| --- | --- | --- | --- |
| **Low** | - Stage IA endometrioid + low-grade + LVSI negative or focal | - Stage I–II ***POLE*mut** endometrial carcinoma, no residual disease  - Stage IA **MMRd/NSMP** endometrioid carcinoma + low-grade + LVSI negative or focal | - Stage I/II *POLE*mut cancer; for **stage III *POLE*mut** cancers  - Stage IA (G1-G2) with endometrioid type (**dMMR and NSMP)** and no or focal LVSI |
| **Intermediate** | - Stage IB endometrioid + low-grade + LVSI negative or focal  - Stage IA endometrioid + high-grade + LVSI negative or focal  - Stage IA non-endometrioid (serous, clear cell, undifferentiared carcinoma, carcinosarcoma, mixed) without myometrial invasion | - Stage IB **MMRd/NSMP** endometrioid carcinoma + low-grade + LVSI negative or focal  - Stage IA **MMRd/NSMP** endometrioid carcinoma + high-grade + LVSI negative or focal  - Stage IA **p53abn** and/or non-endometrioid (serous, clear cell, undifferentiated carcinoma, carcinosarcoma, mixed) without myometrial invasion | -Stage IA G3 with endometrioid type (**dMMR and NSMP**) and no or focal LVSI  -Stage IA non-endometrioid type (serous, clear-cell, undifferentiated carcinoma, carcinosarcoma, mixed) and/or **p53-abn** cancers without myometrial invasion and no or focal LVSI  -Stage IB (G1-G2) with endometrioid type (**dMMR and NSMP**) and no or focal LVSI |
| **High–intermediate** | - Stage I endometrioid + substantial LVSI regardless of grade and depth of invasion  - Stage IB endometrioid high-grade regardless of LVSI status  - Stage II | - Stage I **MMRd/NSMP** endometrioid carcinoma + substantial LVSI regardless of grade and depth of invasion  - Stage IB **MMRd/NSMP** endometrioid carcinoma high-grade regardless of LVSI status  - Stage II **MMRd/NSMP** endometrioid carcinoma | -Stage I endometrioid type (**dMMR and NSMP**) any grade and any depth of invasion with substantial LVSI  -Stage IB G3 with endometrioid type (**dMMR and NSMP**) regardless of LVSI  -Stage II G1 endometrioid type (**dMMR and NSMP**) with substantial LVSI  -Stage II G2-G3 endometrioid type (**dMMR and NSMP**) |
| **High** | - Stage III–IVA with no residual disease  - Stage I–IVA non-endometrioid (serous, clear cell, undifferentiated carcinoma, carcinosarcoma, mixed) with myometrial invasion, and with no residual disease | - Stage III–IVA **MMRd/NSMP** endometrioid carcinoma with no residual disease  - Stage I–IVA **p53abn** endometrial carcinoma with myometrial invasion, with no residual disease  - Stage I–IVA **NSMP/MMRd** serous, undifferentiated carcinoma, carcinosarcoma with myometrial invasion, with no residual disease | -All stages and all histologies with **p53-abn** and myometrial invasion  -All stages with serous or undifferentiated carcinoma including carcinosarcoma with myometrial invasion  -All stage III and IVA with no residual tumour, regardless of histology and regardless of molecular subtype |
| **Advanced/ metastatic** | - Stage III–IVA with residual disease  - Stage IVB | - Stage III–IVA with residual disease of any molecular type  - Stage IVB of any molecular type | / |
