# Supplementary material for: The impact of integrated genomic analysis on molecular classifications and prognostic risk stratification in endometrial cancer: a Chinese experience
Source: Front Oncol. 2025 Feb 6;15:1541562. doi: 10.3389/fonc.2025.1541562 (PMC11839450; doi:10.3389/fonc.2025.1541562)
Supplement: Supplementary file 2 [file DataSheet1.docx]

Supplementary Material

**
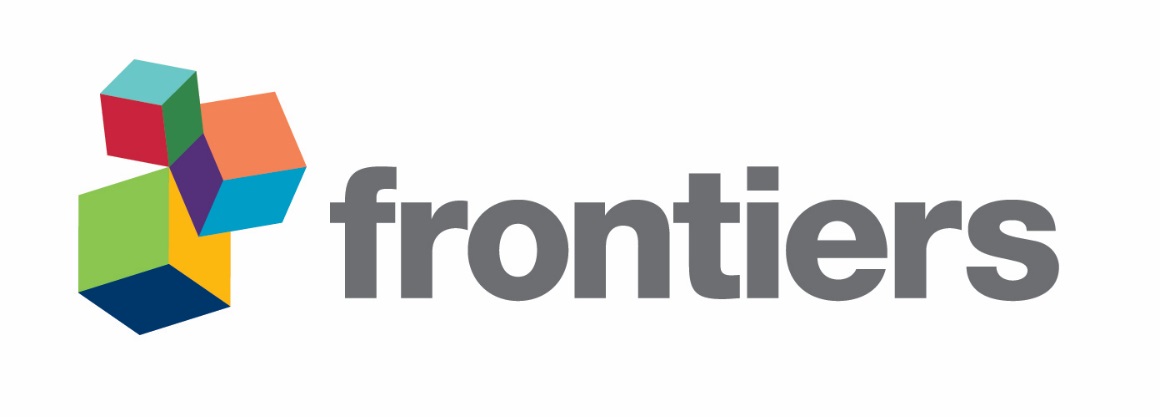
**

**
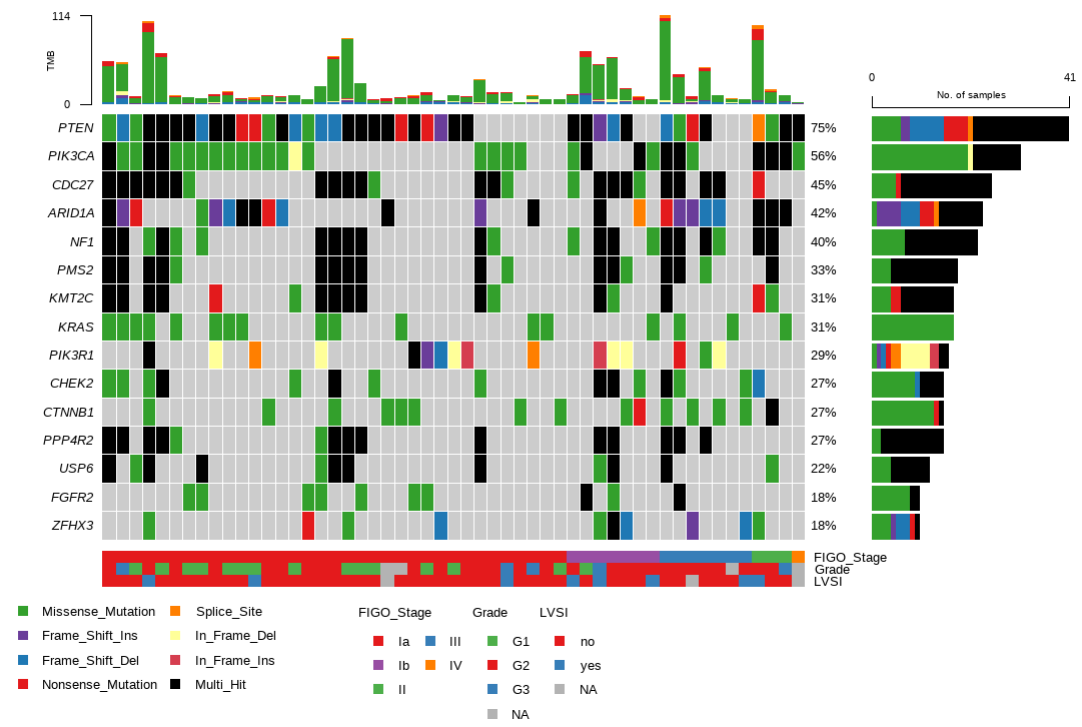
Figure S1** High-Frequency Mutations of Endometrial Cancers in the NSMP Group
